# A 3D flexible neural interface based on a microfluidic interconnection cable capable of chemical delivery

**DOI:** 10.1038/s41378-021-00295-6

**Published:** 2021-08-18

**Authors:** Yoo Na Kang, Namsun Chou, Jae-Won Jang, Han Kyoung Choe, Sohee Kim

**Affiliations:** 1 Department of Medical Assistant Robot, Korea Institute of Machinery & Materials (KIMM), Daegu, Republic of Korea; 2 Center for BioMicrosystems, Korea Institute of Science and Technology (KIST), Seoul, Republic of Korea; 3 Department of Robotics Engineering, Daegu Gyeongbuk Institute of Science and Technology (DGIST), Daegu, Republic of Korea; 4 Department of Brain & Cognitive Sciences, Daegu Gyeongbuk Institute of Science and Technology (DGIST), Daegu, Republic of Korea

**Keywords:** Electrical and electronic engineering, Chemistry

## Abstract

The demand for multifunctional neural interfaces has grown due to the need to provide a better understanding of biological mechanisms related to neurological diseases and neural networks. Direct intracerebral drug injection using microfluidic neural interfaces is an effective way to deliver drugs to the brain, and it expands the utility of drugs by bypassing the blood–brain barrier (BBB). In addition, uses of implantable neural interfacing devices have been challenging due to inevitable acute and chronic tissue responses around the electrodes, pointing to a critical issue still to be overcome. Although neural interfaces comprised of a collection of microneedles in an array have been used for various applications, it has been challenging to integrate microfluidic channels with them due to their characteristic three-dimensional structures, which differ from two-dimensionally fabricated shank-type neural probes. Here we present a method to provide such three-dimensional needle-type arrays with chemical delivery functionality. We fabricated a microfluidic interconnection cable (µFIC) and integrated it with a flexible penetrating microelectrode array (FPMA) that has a 3-dimensional structure comprised of silicon microneedle electrodes supported by a flexible array base. We successfully demonstrated chemical delivery through the developed device by recording neural signals acutely from in vivo brains before and after KCl injection. This suggests the potential of the developed microfluidic neural interface to contribute to neuroscience research by providing simultaneous signal recording and chemical delivery capabilities.

## Introduction

With the development of micromachined neural interfaces, various multifunctional neural interfacing systems have been introduced for different applications^1–5^. Multifunctional neural interfaces not only record neural signals but also directly detect electroactive molecules^6–8^, optically stimulate neurons^9–11^, or deliver drugs by integrating microfluidic channels^12,13^. Direct intracerebral drug injection using microfluidic neural interfaces is an effective way to deliver drugs to the brain, as the blood–brain barrier (BBB) and blood–cerebrospinal fluid barrier prevent the effective delivery of drugs, including anticancer medicines, antibiotics, and anti-inflammatory reagents, to the brain^14^. To date, a number of microfluidic neural interfaces for direct drug injection have been successfully developed using two-dimensional fabrication processes^15–17^, and the possibility of therapeutic uses to treat brain disorders has been demonstrated^18,19^.

Chen et al. developed a neural probe with multiple flow channels and recorded neural signals evoked by various chemical stimuli in guinea pigs^12^. Takeuchi et al. fabricated a parylene-C (PPX-C)-based flexible neural probe integrated with microfluidic channels^20^, which was inserted into a rat brain with the help of supporting materials such as polyethylene glycol (PEG). Altuna et al. presented a SU-8-based microprobe for neural signal recording and drug delivery^21^. Pongracz et al. developed a 70-mm-long silicon multielectrode integrated with microfluidic channels for deep brain research^22^. Rubehn et al. presented a polyimide-based electrode integrated with a SU-8-based waveguide and a microfluidic channel^23^. In their work, an adeno-associated virus was injected using the microfluidic channel, and the light was transmitted through the waveguide, demonstrating successful optogenetic stimulation and neural signal recording in mice. Lecomte et al. experimented with a PEG and silk fibroin-coated PPX-C neural probe^24^. Lee et al. developed a microdialysis probe for neurochemical monitoring^25^. McCall et al. described a wireless optofluidic neural probe that can be utilized for in vivo pharmacology and optogenetics^26^. Shin et al. demonstrated a multifunctional, multishank neural probe that was integrated with an optical waveguide and microfluidic channels; it covered a wide range, including the hippocampal CA3 to CA1 regions in mice, with no significant tissue damage^27^. The functionality of the probe was confirmed by modulating the functional connections of the hippocampus.

All of these microfluidic neural interfaces have employed shank structures that are fabricated in two-dimensional planes, in which microfluidic channels are integrated horizontally along with the shank. The purposes of drug delivery through such shank-type neural probes have been mainly to provide multifunctional tools for neuroscience research, for instance, for the discovery of neurophysiological and neuropathological mechanisms and neural circuits in small animals^28,29^. To accomplish such purposes, it has been important to establish how many channels can be realized in a small area of the shank and how to deliver drugs to a focused target region precisely^12^.

Unlike the aforementioned shank-type neural probes, there are neural interfaces comprised of a collection of microneedles in an array, including, for example, the Utah electrode array (UEA). The UEA is the only microelectrode array that has been approved by the FDA for use with humans^30^ and has been widely used in large animals such as primates because it contains a large number of electrodes to cover a wide region of the brain^31^. The UEA has a characteristic structure in which microneedles are positioned perpendicularly to the array base, unlike the shank-type probes, in which the electrodes are positioned horizontally along the shank. It has been challenging to integrate additional functions into such silicon-based 3-dimensionally fabricated arrays. Therefore, there have been few studies on multifunctional needle-type arrays reported thus far^32,33^. Abaya et al. introduced an optrode array for infrared neural stimulation^34,35^, which is a rare example of studies integrating additional functions into this needle-type electrode array.

Simeral et al. reported the implantation of UEAs with which neural signals were recorded for 2.7 years in humans^36^. At the end of the study, ~60% of the electrodes (56 out of 96) detected spike signals. Despite successful long-term recordings, more than 40% of the electrodes were reported to lose their functionality due to inflammatory responses leading to chronic electrode encapsulation^37^. It has been shown that using shank-type neural probes, the employment of anti-inflammatory drugs such as dexamethasone or neurotrophic medium effectively reduces immunoreactivity during initial immune responses^38–40^. The delivery of anti-inflammatory drugs not only decreases immune responses but also increases the neuronal density around the electrodes^41^. Therefore, adding the capability of drug delivery to the electrodes could improve the lifespans of needle-type neural interfaces, which inevitably involve wounds, or keep more electrodes functional over the entire period of implantation. However, the integration of the drug delivery function into such needle-type microelectrodes has barely been investigated, as it is challenging to integrate a microfluidic channel into the silicon-based 3-dimensional array structure, with needles standing perpendicularly on the wafer surface where fabrication processes are carried out.

**Fig. 1:**
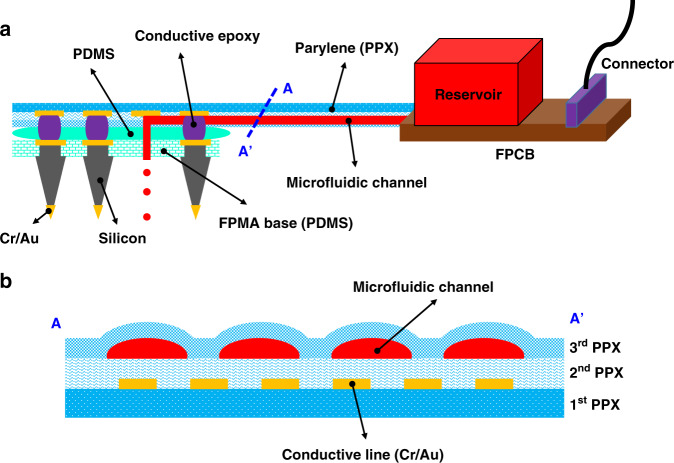
Schematic illustrations of the microfluidic neural interface. **a** The flexible penetrating microelectrode array (FPMA) is integrated with the microfluidic interconnection cable (µFIC). **b** The cross-sectional view of the µFIC (along A-A′) shows that it consists of three layers of parylene-C, with embedded conductive lines and microfluidic channel

Here we present a method for providing three-dimensionally fabricated needle-type arrays with chemical delivery functionality for the first time. We previously reported a flexible penetrating microelectrode array (FPMA), which was fabricated three-dimensionally through dicing and etching of bulk silicon from the beginning of fabrication processes; the device was similar in appearance to the UEA but utilized a flexible array base^42,43^. We also reported a flexible interconnection cable used to connect the FPMA with recording equipment^42,44,45^. In the present study, we propose a 3-dimensional, flexible, and multifunctional neural interface based on an FPMA integrated with a PPX-C-based microfluidic interconnection cable (µFIC) to deliver chemicals to the electrodes (Fig. 1a). The strategy here is to integrate the microfluidic channel into the flexible interconnection cable instead of fabricating the microfluidic channel inside the electrode itself. The fluid flows along the surfaces of needles from the base, resulting in fluid delivery directly to the brain surface but indirectly to the electrodes. The PPX-C-based fabrication of µFICs not only maintains the flexibility of the FPMA but also allows freedom in designing the number, shape, and size of microfluidic channels. Using the developed microfluidic neural interface, we demonstrated chemical delivery capability in combination with recording capability through in vivo acute experiments.

## Materials and methods

### Flexible penetrating microelectrode array (FPMA)

**Fig. 2:**
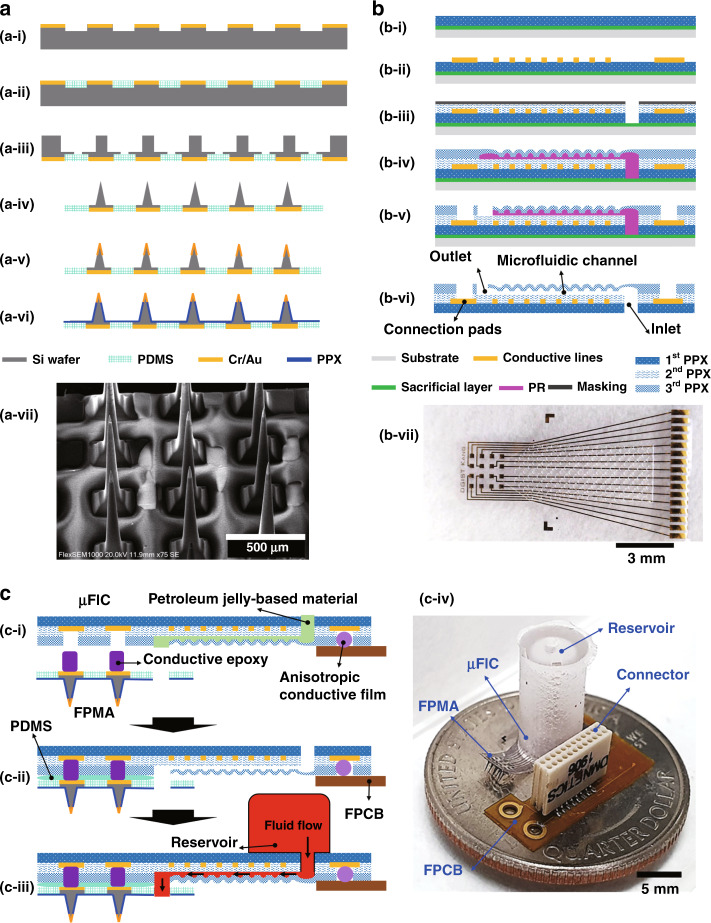
Fabrication procedures for the microfluidic neural interface. **a** Processes used to fabricate the FPMA and a SEM image of the fabricated FPMA. **b** Processes used to fabricate the µFIC: (**b**-i) deposition of a sacrificial layer and the bottom PPX-C layer, (**b**-ii) patterning of conductive lines and pads, (**b**-iii) insulation of conductive patterns by the second PPX-C layer and opening of the inlet of the microfluidic channel using RIE, (**b**-iv) PR patterning and reflow, and deposition of the third PPX-C layer, (**b**-v) RIE to open the outlet of the microfluidic channel and the connection pads, (**b**-vi) release from the wafer and removal of the PR that filled the microfluidic channel. The completed structure of the µFIC is illustrated in (**b**-vi), along with the optical image of the µFIC in (**b**-vii). (**c**-i) to (**c**-iii) Processes to integrate the FPMA, µFIC, and reservoir. (**c**-iv) Fully assembled microfluidic neural interface consisting of the μFIC, FPMA, reservoir, and FPCB

The FPMA used in this study was an array of 4 × 3 electrodes with a flexible polydimethylsiloxane (PDMS) base. The height of silicon needles and the pitch between them were designed to be 1100 and 550 µm, respectively. The FPMA was fabricated as reported in the previous studies^42,43,46^. Figure 2a shows the procedures used to fabricate the FPMA and a scanning electron microscopic (SEM) image of the fabricated FPMA. The tips of the silicon needles were coated with Cr/Au at thicknesses of 25/200 nm (SRN-110, Sorona, Pyeongtaek, Korea). In addition, an insulation layer of PPX-C was deposited at a thickness of 3 µm on the surface of the entire array (NRPC-500, NURI TECH, Incheon, Korea) except for the needle tips^44^. This PPX-C layer helps prevent the silicon needles from being separated from the PDMS base, although the adhesion between the silicon needles and the PDMS base was confirmed previously^42^.

### Microfluidic interconnection cable (µFIC)

Figure 1a presents the schematic drawing of the proposed microfluidic neural interface based on the µFIC, which consisted of three layers of PPX-C, as detailed in Fig. 1b. The first layer was the bottom layer serving as the substrate of conductive metal lines, and inlets were located therein. The width and spacing of the conductive metal lines were 90 and 70 µm, respectively. We created five inlet holes with a diameter of 320 µm for secured infusion into the fluidic channel. The second layer was for the insulation of the conductive metal lines and, at the same time, served as the bottom of the microfluidic channel. A microfluidic channel was formed between the second PPX-C layer (insulation layer) and the third PPX-C layer (microfluidic layer). The µFIC had multiple crater-shaped structures designed to prevent the collapse of the third PPX-C layer^47^. These crater-shaped structures on top of the cable, as illustrated in Fig. 1b, were formed by photoresist (PR) patterning on the second PPX-C layer, which was rounded after reflow at a later step, and deposition of the third PPX-C layer. The microfluidic channel was formed where the PR had been after the removal of the PR. The µFIC had connection pads on the first PPX-C layer to attach the FPMA and a flexible printed circuit board (FPCB) at each end of the cable, as shown in Fig. 2b-vi.

Figure 2b shows the procedures used for the fabrication of the μFIC. First, Ti was deposited at a thickness of 200 nm as a sacrificial layer on a 6-inch silicon wafer using a metal sputtering system (SRN-110, Sorona, Anseong, Korea). The bottom PPX-C layer was deposited at a thickness of 6 µm using a low-pressure chemical vapor deposition (LPCVD) system (NRPC-500, NURI-TECH, Incheon, Korea) (Fig. 2b-i). Cr/Au with thicknesses of 25/200 nm was deposited and patterned by photolithography and etch-back processes to form conductive lines and pads (Fig. 2b-ii). The 3-µm-thick second PPX-C layer was deposited for the insulation of conductive lines and pads. To create inlet holes, a 200-nm-thick Ti layer was patterned as a mask on the insulation layer. Oxygen plasma etching was carried out using a reactive ion etching (RIE) system (FabStar, Top Technology, Hwaseong, Korea) with 300 W power, 100 sccm oxygen flow rate, and 500 mTorr pressure (Fig. 2b-iii). After the removal of the Ti mask, a photoresist (AZ®40XT, Microchemicals, Ulm, Germany) was patterned on the second PPX-C layer to create the microfluidic channel. The PR pattern was reflowed on a hot plate at 130 °C to form the arch-shaped fluidic channel and crater-shaped structures, which were removed to create the space through which fluids could flow at the last fabrication step. The top layer of the microfluidic channel was formed by the third PPX-C layer, which was deposited at a thickness of 6 µm (Fig. 2b-iv). To expose the pads and to create the outlet of the microfluidic channel, RIE was conducted using O_2_ plasma with a flow rate of 80 sccm, pressure of 200 mTorr, and power of 300 W (Fig. 2b-v). Finally, the µFIC was released from the silicon wafer and immersed in acetone and isopropyl alcohol for 5 min each, to remove the PR pattern that filled the inside of the channel (Fig. 2b-vi), resulting in a highly flexible interconnection cable integrated with the arch-shaped microfluidic channel (Fig. 2b-vii).

### Assembly and packaging

The fabricated μFIC was assembled with the FPMA, a custom-designed FPCB, and a drug reservoir, as shown in Fig. 2c. The µFIC had two sections of connection pads to attach the FPCB and the FPMA at both ends. First, the connection pads on the µFIC and the FPCB were aligned using optical microscopy and connected through an anisotropic conductive film (ACF) (AC-7246LU-18, Hitachi Chemical, Tokyo, Japan) using an ACF bonding machine (ZH-012, Shenzhen ZWX Technology, Guangdong, China) (Fig. 2c-i). The polyimide-based FPCB was used to enable secure connection and disconnection of the µFIC and the recording system. Next, the FPMA was attached to the pads at the other end of the µFIC using conductive epoxy (125 epoxy kit, DuralcoTM, New York, NY, USA) (Fig. 2c-i). The epoxy was cured at room temperature for 24 h to prevent thermal oxidation of the PPX-C-based µFIC^48–50^. Before the attachment of the FPMA, one of the needles on the FPMA was removed by pulling out rapidly with forceps to create the outlet of the microfluidic channel. The place where the needle was removed was aligned to the outlet of the microfluidic channel. After curing the conductive epoxy, PDMS was filled into the space formed between the FPMA and the µFIC to maintain the electrical independence of each electrode even in an implanted environment (Fig. 2c-ii). To prevent PDMS from flowing into the outlet while filling the PDMS, the outlet of the µFIC was protected with a petroleum jelly-based material (Vaseline^®^, Unilever, London, UK), which melts at body temperature. After the filled PDMS was cured, the outlet of the µFIC was opened again by absorbing the jelly-based material using oil paper in an oven at 37 °C (Fig. 2c-ii). At the other end of the FPCB, an Omnetics connector (A79018-001, Omnetics, Minneapolis, MN, USA) was soldered, as shown in Fig. 2c-iv.

**Fig. 3:**
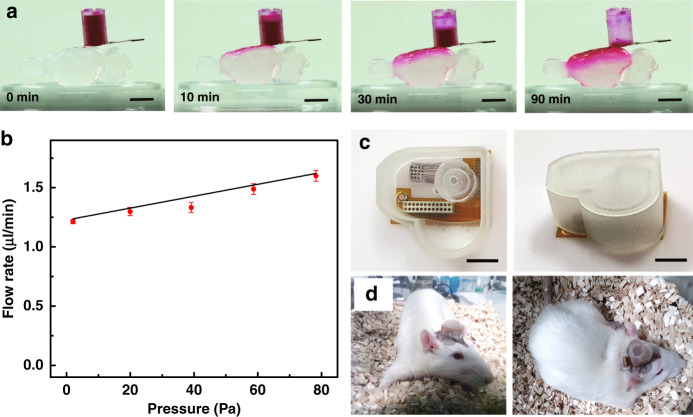
Fluid delivery through the developed microfluidic neural interface and its implantation in a rat. **a** Sequential images show the delivery of colored water to a brain model made of 1% agarose. The colored water diffused into the agarose over time. **b** Flow rate through the microfluidic neural interface, as a function of pressure. Red dots and error bars represent means and standard deviations, respectively, while the black line represents linear fitting. Photographs of **c** the custom-designed head cap and **d** a rat implanted with the microfluidic neural interface protected by the head cap. (scale bar: 5 mm)

The drug reservoir was designed to be cylindrical in shape, with a total volume of ~120 µl. It was made of PDMS and coated with a 3-µm-thick layer of PPX-C to prevent the evaporation of the fluid contained in it and to make the surface of the reservoir hydrophobic. To release the internal pressure, a hole with a diameter of 1 mm was drilled in the top of the reservoir. Before attachment, oxygen plasma was treated to the drug reservoir in order to increase the adhesion between the drug reservoir and the µFIC. The drug reservoir was integrated onto the µFIC using UV curable epoxy (Fig. 2c-iii). To prevent the flow of UV curable epoxy into the inlet, the microfluidic channel was filled temporarily with PEG, which is solid at room temperature but melts at body temperature. PEG was removed by soaking in deionized water at 37 °C after the drug reservoir was attached to the μFIC. Finally, PDMS was applied to provide mechanical durability and solid electrical insulation at every coupling part where the μFIC and the FPCB were connected and where the Omnetics connector and the FPCB were connected. To protect the fully assembled microfluidic neural interface implanted on an animal’s brain, a custom-designed head cap was fabricated with 3D printing using a multi-material rapid prototyping machine (Projet MJP 5500X, 3D systems, Rock Hill, SC, USA), as shown in Fig. 3c.

### In vivo experimental procedures

All experimental procedures were approved by the Institutional Animal Care and Use Committee at the Daegu Gyeongbuk Institute of Science & Technology (DGIST) Laboratory Animal Resource Center (approval number: DGIST-IACUC-20011506-07). Five adult male Sprague-Dawley rats (Daehan Biolink, Eumseong-gun, Korea) weighing 280–320 g were used. Among the five, three were used for chemical delivery and neural recording experiments, and two were used for histological analysis.

All animals were single-housed under a 12 h light/dark cycle with free access to food and water and at a controlled temperature of 22–25 °C. The animals were acclimatized to the animal care facility for a week prior to surgery^51^. Before the surgery, the animals were deeply anesthetized using a respiratory anesthetic system (MatrxTM VIP 3000, Midmark, Dayton, OH, USA). Anesthesia was induced in a chamber with 5.0% isoflurane for 3 min and maintained with inhalation of 2.0–2.5% isoflurane through a mask. The animals were mounted on a stereotaxic frame (KOPF #902, David Kopf Instruments, Tujunga, CA, USA) during the experiment. After incision of the skin, a hole with a diameter of ~3.5 mm was drilled through the skull for implantation of the fabricated microfluidic neural interface onto the motor cortex. The center of the hole was placed at the primary motor cortex (anterior-posterior (AP): 1.75 mm, medial-lateral (ML): 2.00 mm from bregma^52^). After the dura mater was removed carefully, the electrodes were implanted under instantaneous pressure using a pneumatically actuated impulse inserter (Research inserter IFU, Blackrock^®^ Microsystems, Salt Lake City, UT, USA) to minimize misalignment between the needles of the FPMA in the cortex. The headstage of the recording system was connected with the microfluidic neural interface, and the wand of the pneumatic inserter was mounted on the stereotaxic frame to allow stable operation without shanking. Then, the implanted microfluidic neural interface was secured on the brain using a silicone elastomer (Kwik-Sil, World Precision Instruments, Sarasota, FL, USA) and protected by the head cap fixed on the skull using screws and dental cement, as shown in Fig. 3d.

### Neural signal acquisition and chemical delivery

After the surgery, the anesthesia treatment of the rats was maintained. Spontaneous neural signals were recorded at a sampling rate of 30 kHz using a digital recording system (CerePlex Direct, Blackrock^®^ Microsystems, Salt Lake City, UT, USA). Prior to the experiments for fluid delivery, neural signals were recorded using the microfluidic neural interface. First, signals were recorded for 120 s every 10 min for a total period of 20 min without any treatment for baseline. Then, 120 μl of 10 mM KCl in a solution of artificial cerebrospinal fluid (aCSF), prepared following a standard protocol^23,53^, was infused into the reservoir. Neural signals were recorded for 120 s every 10 min for a total period of 30 min immediately after the KCl solution was injected into the reservoir. After recording, the acquired raw neural signals were bandpass filtered from 250 to 5000 Hz and used for spike sorting. Blackrock Offline Sorting Software and CerePlex Direct Software (Blackrock^®^ Microsystems, Salt Lake City, UT, USA) were used to detect spikes and calculate signal-to-noise ratios (SNRs). The raw data were then extracted and converted into a format compatible with Spike 2 (Cambridge Electronic Design, Cambridge, UK) by MATLAB (The MathWorks Inc., Natick, MA, USA). Spike sorting and drawing of raster plots were performed using Spike 2. Waveform data were manually discriminated using visual features within 1.6 ms when the signal amplitude exceeded an adjusted threshold. The extracted waveforms from Spike 2 were plotted again using Origin (OriginLab Co., Northampton, MA, USA).

To verify the region of delivery around the electrodes, microfluidic neural interfaces were implanted into two rats, and Dextran Texas Red^TM^ (Invitrogen^TM^, Waltham, MA, USA) was injected. A total volume of 20 µL was injected with a flow rate of 1.2 to 1.5 µL/min. After the injection, the animal was left for 10 min for the fluid to diffuse. Then, the animal was sacrificed and perfused with phosphate-buffered saline (PBS) and 4% paraformaldehyde (PFA) solution sequentially with a flow rate of 14 mL/min for 20 min^54^. The extracted brain was sliced with a thickness of 100 µm using a vibratome (VT1200s, Leica Biosystems, Wetzlar, Germany), and slices were stained with DAPI to label the nuclei of cells.

## Results and Discussion

### µFIC and fully integrated microfluidic neural interface

**Fig. 4:**
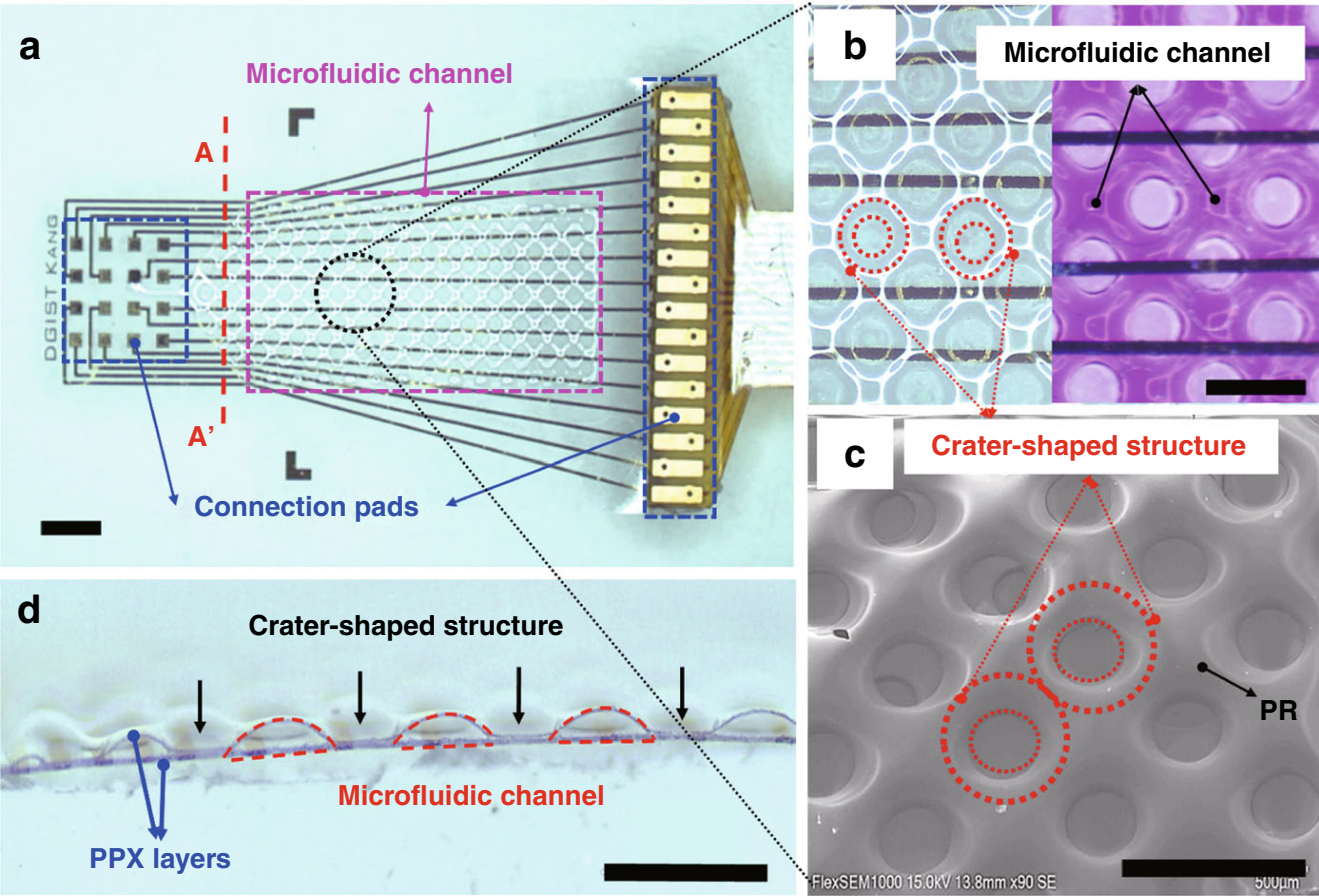
Optical images of the μFIC. **a** Top view of the μFIC. The μFIC has a microfluidic channel and connection pads to attach the FPMA (left) and the FPCB (right) (scale bar: 1 mm). **b** The magnified views show when the microfluidic channel is empty (left) and filled with dyed water (right). **c** SEM image of the μFIC. The μFIC has crater-shaped structures that help prevent the collapse of the third PPX-C layer. **d** Cross-sectional view of the μFIC showing the arch-shaped microfluidic channel. The width and height of the channel are ~200 and 40 µm, respectively (scale bar: 500 µm in **b**–**d**)

The fabricated µFIC is shown in Fig. 4a. The µFIC was 13 mm long, 8 mm wide, and had multiple crater-shaped structures to support the third layer of PPX-C. The shape of the microfluidic channel depended on the PR pattern created by the reflow process. The PR pattern had multiple holes with a diameter of 200 µm and a distance between the centers of 500 µm. The sharp edges of the PR pattern turned into rounded shapes during the reflow process.

The holes in the PR pattern were deposited with PPX-C, becoming crater-shaped structures in the final form, as shown in Fig. 4b, while the volume occupied by the PR became the inside of the microfluidic channel after removal of the PR (Fig. 4c). These crater-shaped structures were located in a zig-zag fashion (Fig. 4b) and thus, helped to avoid collapse or folding of the microfluidic channel by supporting the top PPX-C layer. The crater-shaped and zig-zag-placed structures were also used to slow fluid flow in the channel, compared to flow in channels with no such structure or with in-line positioned structures^55^. The cavity generated by removing the PR between the second and third PPX-C layers was the passage through which the fluid flowed. Figure 4d shows an optical image of the cross-section of the µFIC. The microfluidic channel had arch-shaped cross-sections with a maximum height of ~40 µm. The number and size of the microfluidic channel depend on the designed PR pattern, which can be easily modified if necessary. Although a microfluidic channel was fabricated in the present study to verify the function of chemical delivery to the FPMA for the first time, it is expected that up to four channels could be fabricated with a minimum channel width of 50 µm, taking into account the size of the inlet and the entire dimension of the µFIC.

To create the outlet, we removed one of the needles, and then, the outlet of the microfluidic channel was aligned at the location from which the needle was removed. Although the outlet was created at the perimeter of the array in the current study, it can be made in the central region as well. The fully integrated microfluidic neural interface, including the FPMA, the µFIC, and the reservoir, with dimensions of 12.5 mm × 16 mm × 8 mm (W × L × H), is shown in Fig. 2c-iv. The custom-designed head cap, which is shown in Fig. 3c, was used to protect the microfluidic neural interface after implantation in animals.

### Characterization of fluid flow through the µFIC

To evaluate fluid delivery through the fabricated µFIC, the flow rate was characterized (Fig. 3a). A rat brain model made of 1% [w/v] agarose gel, which was slightly denser than brain tissue, was used^56^. Using dyed water, we observed passive flow into the model brain through diffusion. The flow rate was calculated based on the total volume of the injected fluid and the time taken. Fig. 3b shows the measured flow rate through the µFIC, which was 1.40 ± 0.15 µL/min (mean ± standard deviation). The maximum allowable flow rate through the µFIC was observed to be 5 µL/min^57^. Typically, the total volume of cranial CSF in adult rats is known to be 275 to 440 µL, and the reproduction rate is 1.77 to 2.84 µL/min^58^. The typical flow rate for direct intra-CSF infusion into the rodent brain is approximately up to 2 µL/min^59,60^. Considering those previous results, the measured passive flow rate was within an acceptable range.

### In vivo recording and chemical delivery

**Fig. 5:**
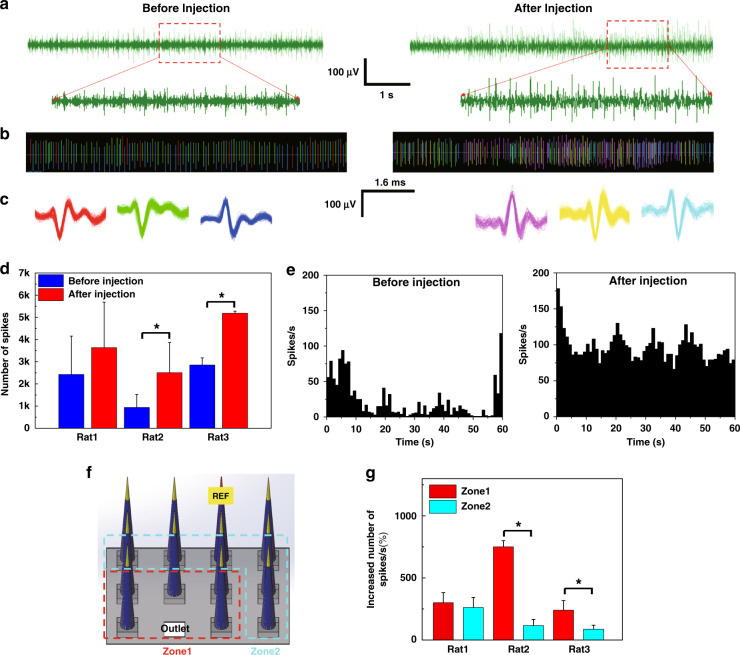
Neural signals recorded by the microfluidic neural interface before and after the injection of 10 mM KCl solution to in vivo rat brains. **a** Representative examples of raw signals recorded by an electrode before and after KCl injection. **b** Raster plots of sorted spikes with **c** distinctive spike waveforms. **d** The numbers of spikes seen during 120 s recordings before and after injection. After injection, the number of spikes increased by ~80% on average. In rats 2 and 3, the numbers of spikes were significantly different before and after injection. **e** The number of spikes per second from a representative electrode before and after injection. **f** Divided sections in the FPMA according to the distance from the outlet. **g** Increase in the number of spikes after injection in zones 1 and 2. In rats 2 and 3, the difference between zones 1 and 2 was significant while no significant difference between zones was observed for rat 1. The error bars represent standard deviations. An asterisk (*) indicates *p* < 0.05 (one-way ANOVA and Bonferroni test)

The fabricated microfluidic neural interface was validated through in vivo acute experiments to demonstrate both electrical recording and chemical delivery. We recorded the spontaneous neural activity of the primary motor cortex using the device. The in vivo impedance from all 33 electrodes in three FPMAs was measured to be 374.32 ± 36.86 kΩ (mean ± standard deviation). One of the electrodes in each FPMA was assigned as the reference electrode, as shown in Fig. 5f. Spike signals were detected with more than six out of ten electrodes: seven electrodes in rat 1, eight electrodes in rat 2, and six electrodes in rat 3. Figure 5a shows representative raw signals obtained by one of the electrodes implanted in rat 2 before and after KCl injection.

Chemical depolarization typically requires a 20 min injection of potassium solution, possibly due to the delivery and diffusion required to reach an effective concentration, to induce changes in electric activity. Considering the lag time of the chemical depolarization protocol, signals measured at 30 min after injection were analyzed. From the 21 functional electrodes in the three implanted arrays, the averaged background noise levels were 43.4 and 37.8 µV_PP_ before and after injection, respectively. Spikes were detected from multiple neurons by a single electrode, with amplitudes of 114.9 ± 9.4 µV_PP_ (mean ± standard deviation) before injection and 129.7 ± 8.8 µV_PP_ after injection. Therefore, the SNR was calculated to be 2.9 ± 0.5 and 3.5 ± 0.7 before and after injection, respectively. Figure 5b shows the difference between firing rates measured before and after injection. The raster plots derived from spike sorting confirmed that each individual neuron was sorted. In Fig. 5c, the representative waveforms detected by an electrode implanted in rat 2 are compared before and after injection. After injection, the signal amplitude tended to increase slightly, and even changes in waveform were observed, which would indicate that, due to the increased signal amplitude, spike signals from neurons that were not detectable before injection could be detected after injection.

Figure 5d shows the number of spikes in three rats before and after injection. The number of spikes detected by each electrode was counted from neural signals recorded for 120 s at 20 min before injection and at 30 min after injection. The bar graph represents the average number of spikes of all electrodes in each array, with error bars indicating standard deviations. In all animals, an increasing tendency in the number of spikes was consistently detected after KCl injection, as expected. Data were further analyzed by one-way ANOVA followed by the Bonferroni post hoc test to determine statistical significance (*p* < 0.05). In rats 2 and 3, a significant elevation in the frequency of spikes was observed by all electrodes after injection. Fig. 5e shows a comparison of the numbers of spikes per second before and after injection using one of the electrodes implanted in rat 3. The number of spikes clearly increased after injection compared to that before injection. Thus, the capability of the developed microfluidic neural interface to deliver chemicals was confirmed by simultaneous recording of neural signals.

Next, the number of spikes as a function of distance from the outlet was compared. The microelectrodes were divided into two sections: electrodes adjacent to the outlet (zone 1) and the rest (zone 2), as shown in Fig. 5f. Figure 5g shows an increased percentage in the number of spikes per second after chemical injection. A significant difference (*p* < 0.05) between zones 1 and 2 was observed in rats 2 and 3. Therefore, it can be concluded that the distance-dependent difference in chemical delivery was significant even with passive diffusion-based delivery.

**Fig. 6:**
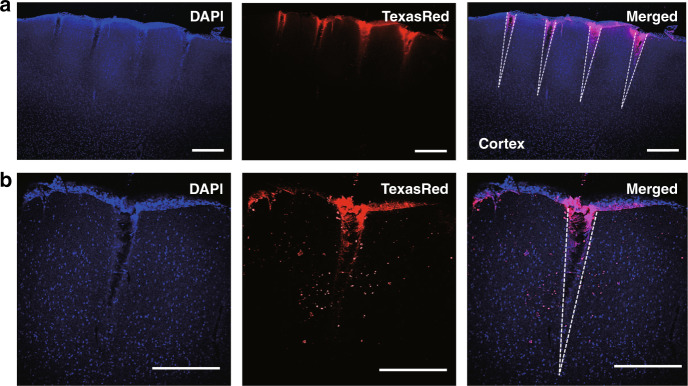
Histological examination of fluid delivery to the brain in vivo. **a** Fluorescence images of the rat brain section containing the electrode implantation site. DAPI and Dextran Texas Red were used to label cell nuclei and to visualize the region of delivery, respectively. **b** Magnified images of the area around an electrode. The white dotted lines represent electrode tracks. (scale bar: 500 µm)

We then histologically examined the range of chemical diffusion around the electrodes. Texas Red-conjugated dextran was injected for 10 min through the outlet located at the base of the microfluidic neural interface to label cells within the effective diffusion range. In Fig. 6, nuclei of cells (blue) and diffused Texas Red-dextran-labeled cells (red) are found in the same area of the brain. The dextran-labeled cells were located within and in close proximity to the cavity of the sharp V-shaped notch formed by microneedle placement, indicating that Texas Red-dextran flowed along the electrodes and diffused into the brain tissue. Histological examinations confirmed that the microfluidic neural interface presented here is capable of delivering chemicals to the electrode sites.

## Conclusions

We presented a three-dimensional flexible and multifunctional neural interface, in which a FPMA was integrated with a µFIC. It demonstrated the capability of fluid delivery and simultaneous recording of neural signals in in vivo animals. In the present study, fluid delivery was based on diffusion, and thus, the region more distant from the outlet was influenced less by the delivered chemical, implying that the distant regions would be influenced at a later point in time. To the best of our knowledge, this is the first microfluidic neural interface to add a chemical delivery capability to three-dimensional electrode arrays comprising a collection of microneedles positioned perpendicular to the array base, including the FPMA and the UEA. Moreover, thanks to the flexibility of the device, the developed microfluidic neural interface is expected to cover a large area of the brain even with curvatures, which suggests its potential usage in nonhuman primates and even in humans who have large and convoluted brains. Our study also suggests the possibility of delivering anti-inflammatory drugs to needle-type electrode arrays used for long-term implantation. In addition, the successful recording of spike signals before and after chemical delivery indicates that the developed microfluidic neural interface could be used to evaluate drugs to treat brain dysfunctions. As a future study, the delivery of anti-inflammatory drugs for a long-term period needs to be investigated.
